# In Vitro Comparative Effects of Pulpine Mineral, Pulpine NE, and MTA on the Viability, Proliferation, Migration, and Attachment of Stem Cells from Human Exfoliated Deciduous Teeth: In Vitro Comparative Effects of Pulpine Mineral, Pulpine NE, and MTA on Stem Cells from Human Teeth

**DOI:** 10.31661/gmj.v13iSP1.3714

**Published:** 2024-12-31

**Authors:** Masumeh Moslemi, Maryam Torshabi, Mahta Khosrozamiri

**Affiliations:** ^1^ Department of Pediatric Dentistry, School of Dentistry, Shahid Beheshti University of Medical Sciences, Tehran, Iran; ^2^ Department of Dental Biomaterials, School of Dentistry, Shahid Beheshti University of Medical Sciences, Tehran, Iran

**Keywords:** Adult Stem Cells, Cell Adhesion, Cell Migration, Cell Survival, Dental Cements

## Abstract

**Background:**

This research aimed to evaluate the impact of Pulpine Mineral, Pulpine NE, and mineral trioxide aggregate (MTA) Angelus on crucial cellular functions, including viability, proliferation, attachment, and migration, in stem cells derived from human exfoliated deciduous teeth (SHEDs).

**Materials and Methods:**

In this laboratory-based investigation, SHEDs were exposed to 24-hour extracts from Pulpine Mineral, Pulpine NE, and MTA Angelus, prepared in both freshly mixed and fully set forms, over 24 and 72 hours. The methyl thiazolyl tetrazolium (MTT) assay was used to measure cell viability and proliferation, while a scratch test assessed the extent of cell migration. Scanning electron microscopy (SEM) provided insights into how these materials affected cell morphology and attachment. Data analysis was performed using one-way ANOVA and Tukey’s post-hoc test, with statistical significance set at α=0.05.

**Results:**

Among the materials tested, MTA resulted in significantly greater cell viability than the other groups (P0.05). Interestingly, diluted extracts of set Pulpine Mineral showed comparable viability to MTA after 24 hours (P0.05). In contrast, Pulpine NE yielded the lowest viability scores (P0.05). For migration, the MTA group achieved complete scratch closure within 48 hours, whereas Pulpine Mineral facilitated partial migration but did not close the scratch entirely. Cells in the Pulpine NE group exhibited neither proliferation nor migration, as they were entirely non-viable.

**Conclusion:**

Pulpine Mineral showed superior biological effects compared to Pulpine NE; however, both Pulpine materials exhibited inferior results compared to MTA Angelus.

## Introduction

Vital pulp therapy (VPT) includes a range of conservative techniques [1] aimed at regenerating the dentin-pulp complex that may be damaged by dental trauma, caries, accidental injuries, or during restorative procedures [2]. It is recommended for cases where the pulp shows signs of reversible or even irreversible damage, provided no periapical lesions are present [3]. Among VPT methods, indirect pulp capping is the least invasive, followed by direct pulp capping and pulpotomy [3][4].

VPT is a preferable alternative to root canal therapy wherever feasible because root canal treatment removes potentially viable pulp tissue and often leads to periapical tissue responses to the root-filling materials after pulp removal. Conversely, VPT promotes a natural physiological response and offers biological therapeutic benefits [5]. The procedure involves applying pulp capping agents that encourage the formation of a protective mineral layer over the dentin-pulp complex. This approach preserves tooth vitality and fosters a biocompatible environment conducive to tissue regeneration and healing [6].

Mineral trioxide aggregate (MTA) has long been regarded as the gold standard for VPT due to its beneficial biological features, including biocompatibility, bioactivity, low solubility, and hydrophilic nature [7]. Despite these advantages, MTA has certain drawbacks, such as challenging handling, extended setting times, discoloration, and a sandy texture [8]. Even so, MTA, as a Portland cement-based material, has inspired the development of newer bioceramic and calcium silicate-based materials with enhanced biological properties and antibacterial effects [9].

Certain organic compounds have been proposed as alternatives to synthetic materials for use in VPT. Among them, propolis—a resinous substance collected by honeybees from plant sources has shown potential health benefits. In dental applications, propolis has been utilized as an intracanal medicament, a cariostatic agent, a storage medium for avulsed teeth, and an intracanal irrigant. Its rising popularity is largely attributed to its anti-inflammatory and immunomodulatory properties [10][11][12].

Multiple in vitro studies have evaluated the cytotoxic effects of propolis on human cell lines. Additionally, animal studies involving the ethanolic extract of propolis have reported encouraging results, such as bone regeneration and the induction of hard tissue formation when used for pulpotomy or as a pulp capping agent [13][14][15].

Hoffmann has incorporated propolis as a key ingredient in the liquid component of its two new VPT products, Pulpine Mineral and Pulpine NE, offering a replacement for MTA. Pulpine Mineral comes in a powder-and-liquid formulation, with its powder containing calcium hydroxide and hydroxyapatite, while its liquid combines propolis and ethanol. Similarly, Pulpine NE’s powder includes calcium hydroxide and zinc oxide, paired with the same liquid composition of propolis and ethanol as in Pulpine Mineral [16].

Compared to DPSCs, SHED, a type of multipotent mesenchymal stem/stromal cells (MSCs), demonstrated better osteogenesis-inducing capacity, a faster population doubling time (PDT), a greater proliferation rate, and more immature multipotent cells[17].

The manufacturer has strongly emphasized the favorable characteristics of Pulpine Mineral and Pulpine NE, suggesting they may serve as effective alternatives to MTA, which remains the gold standard in VPT. Consequently, this study sought to evaluate the effects of these materials—Pulpine Mineral and Pulpine NE—on the viability, proliferation, adhesion, and migration of stem cells derived from human exfoliated deciduous teeth (SHEDs), comparing their performance to MTA.

## Materials and Methods

The study protocol was reviewed and approved by the university’s ethics committee of Shahid Beheshti University of Medical Sciences (IR.SBMU.DRC.REC1402.103).

Stem cells were purchased from the Cell Bank of the Research Institute of Dental Sciences.

To evaluate cytotoxicity and cell migration, cells were indirectly exposed to material extracts, whereas direct exposure to the materials was used for assessing cell adhesion.

### Preparation of the Extracts:

Discs measuring 1 cm in diameter and 2 mm in thickness were prepared from MTA Angelus (Angelus, Brazil), Pulpine Mineral (Hoffman, Germany), and Pulpine NE (Hoffman, Germany) in accordance with the manufacturers’ instructions under aseptic conditions. Five discs were fabricated for each material. The discs were sterilized with ultraviolet (UV) light for 30 minutes on each side.

For fresh extract preparation, Dulbecco’s modified Eagle’s medium (DMEM; Gibco, UK) containing 10% fetal bovine serum (FBS; Gibco, UK) was added to the sterilized discs following the ISO-10993-12 guidelines. The discs were then incubated at 37°C in a CO₂ incubator (Binder, Germany) with 95% humidity for 24 hours. To obtain extracts from set specimens, the discs were first allowed to fully set by incubating them under the same conditions for 24 hours. Once set, DMEM containing 10% FBS was added to the discs, again following ISO-10993-12, and they were incubated for an additional 24 hours at 37°C and 95% humidity. The prepared extracts were then refrigerated for future use.

### Assessment of Cell Viability, Proliferation, and Cytotoxicity

The cytotoxicity of MTA, Pulpine Mineral, and Pulpine NE, along with their effects on cell viability and proliferation, was evaluated using the methyl thiazolyl tetrazolium (MTT) assay. Stem cells from human exfoliated deciduous teeth (SHEDs) were seeded in 96-well plates (SPL, Korea) at a density of 5,000 cells per well, each containing 100 µL of Dulbecco’s modified Eagle’s medium (DMEM) with 10% fetal bovine serum and 1% antibiotic. The plates were incubated at 37°C with 95% humidity in a CO₂ incubator for 24 hours to allow the cells to reach the logarithmic growth phase and approximately 70% confluence.

After incubation, the medium was removed and replaced with material extracts in three forms: undiluted (100%), and diluted to 50% and 25%. Cells exposed to the extracts formed the test groups, while cells cultured in medium alone acted as the control group. The plates were incubated again under the same conditions. Acute cytotoxicity was assessed after 24 hours, and chronic cytotoxicity was evaluated after 72 hours using the MTT assay, following the ISO-10993-5 standard.

The assay involved replacing the culture medium in each well with 100 µL of DMEM containing 10% MTT dye (Sigma Aldrich, Germany). The plates were incubated for three hours at 37°C and 95% humidity in a CO₂ incubator, allowing for the formation of purple formazan crystals. Subsequently, the medium was removed, and 100 µL of dimethyl sulfoxide (DMSO) was added to dissolve the crystals. The optical density (OD) of the solution in each well was measured using a microplate reader (ELISA Reader; Anthos 2020) at a wavelength of 570 nm.

Each group in the studies was given a minimum of six repeats, and the experiments were conducted at least twice at various times.

To determine cell viability, the mean OD of each treated group was divided by the mean OD of the control group (assumed as 100% viability) and multiplied by 100.

According to the ISO-10993-5 standard, any material that reduces cell viability by more than 30% compared to the control (reducing viability below 70%) is classified as cytotoxic [18].

### Assessment of Cell Migration Using the Scratch Test

On the first day of the experiment, SHEDs in their logarithmic growth phase were seeded in 24-well plates at a density of 100,000 cells per well. By the second day (24 hours after incubation), the cells reached full confluence (100%), and a vertical scratch was made in each well using the tip of a #10 sterile sampler to create the starting point (time 0). Detached cells from the scratch area were removed by rinsing each well twice with complete culture medium.

Afterward, the wells were treated with either the culture medium alone (control group) or a ¼ concentration of the extracts derived from fully set MTA, Pulpine Mineral, and Pulpine NE specimens. The cells were stained and imaged at the start of the experiment (time 0) and after 24 and 48 hours. Digital photographs were captured using an inverted microscope (Nikon, Japan).

### Cell Staining Procedure

To prepare the cells for imaging, the overlaying medium was first removed, and the cells were rinsed twice with phosphate-buffered saline (PBS) containing cold calcium and magnesium (at 4°C). To fix the cells, 500 µL of pre-cooled methanol (100%, stored at -20°C) was added to each well, followed by incubation at room temperature for 10 minutes. The methanol was then removed, and a 0.5% crystal violet solution (Merck, Germany) was applied to the cells. The plate was placed on a shaker at room temperature for 10 minutes to ensure thorough staining. After removing the staining solution, the cells were rinsed three times with deionized water.

Using an inverted light microscope at 4x magnification, digital images of the cells along the scratch site were captured. The distance between the edges of the scratch was measured in inches using Image J software.

### Assessment of Cell Morphology and Adhesion

After 24 hours of incubation to ensure the specimens were fully set and UV-sterilized, they were placed into wells of a 24-well plate and sealed with agar. SHEDs were then seeded on the specimen surfaces at a density of 50,000 cells per surface. The plates containing the specimens and cells were incubated.

At 24 and 48 hours, the plates were removed from the incubator. The medium was carefully removed, and the specimens were transferred to new wells. Each specimen was rinsed with phosphate-buffered saline, followed by the addition of 500 µL of 2.5% glutaraldehyde in phosphate-buffered saline to fix the cells adhered to the specimen surfaces. The plates were refrigerated for 24 hours to complete the fixation process.

Following fixation, the specimens were rinsed and dehydrated using a series of graded alcohol concentrations in the following sequence: deionized water for 10 minutes, 30% alcohol for 10 minutes, 40% alcohol for 10 minutes, 50% alcohol for 10 minutes, 60% alcohol for 10 minutes, 70% alcohol for 10 minutes, 80% alcohol for 10 minutes, 90% alcohol for 10 minutes, and 100% alcohol for 30 minutes. The alcohol was then removed, and the specimens were air-dried under a chemical hood for 48 hours. Once dried, the specimens were coated with gold using a sputter-coating process and visualized under a scanning electron microscope (SEM).

### Statistical Analysis

Taking into account the homogeneity of variances as demonstrated by the Levene’s test (P>0.05) and the normal distribution of cell viability data as verified by the Shapiro-Wilk test (P>0.05), data from the MTT assay were analyzed using GraphPad Prism (version 9.0.0). General comparisons were performed using one-way ANOVA, followed by pairwise comparisons using Tukey’s test, with the level of significance set at 0.05.

## Results

**Figure-1:**
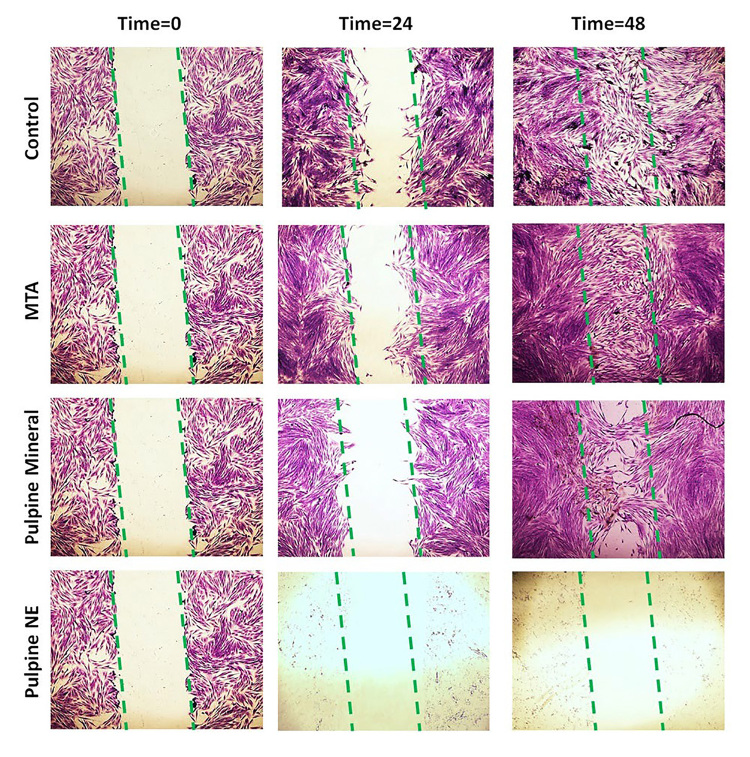
Figure 1. Comparison of the cytotoxicity of MTA, Pulpine Mineral, and Pulpine NE extracts after 24 hours in fresh (A) and set (B) forms. Starts on top of the columns indicate presence of a significant difference with the control group, and starts on the lines indicate presence of a significant difference between the two groups (P< 0.05)

### Results of the MTT assay for MTA cytotoxicity:

After 24 Hours:

There was no significant difference in cell viability among the groups treated with different concentrations of the set MTA extract. These groups also showed no significant difference when compared to the control group (P>0.05), with all displaying approximately 100% cell viability. Similarly, cells exposed to various concentrations of the fresh MTA extract exhibited no significant differences in viability among themselves. However, their viability percentages were approximately 20% lower than those of the control group, a statistically significant reduction (P<0.05).

Overall, cell viability was significantly higher in specimens treated with set MTA extract compared to those treated with fresh MTA extract (P<0.05).

After 72 Hours:

For cells treated with different concentrations of set MTA extract, no significant differences in viability were observed among the groups or when compared to the control group (P>0.05), except for the group treated with pure extract. In this case, a 20% reduction in cell viability was noted compared to the control group, which was statistically significant (P<0.05).

For cells treated with ½ and ¼ concentrations of fresh MTA extract, viability percentages did not differ significantly from one another or from the control group (P>0.05). However, the pure extract caused a significant 80% reduction in cell viability compared to the control group (P<0.05).

### Results of the MTT assay for Pulpine Mineral cytotoxicity

After 24 hours:

No significant difference was found in the viability percentage of cells treated with ½ and ¼ concentrations of set Pulpine Mineral extract with each other or with the control group (P>0.05). However, pure extract caused a significant reduction of approximately 40% in cell viability compared with the control group and diluted extracts (P<0.05). The percentage of reduction in cell viability was approximately 80% in pure extract, approximately 70% in ½ diluted extract, and approximately 40% in ¼ diluted extract of fresh specimens (P<0.05), showing cytotoxicity of the fresh Pulpine Mineral extract. In total, the percentage of cell viability was significantly higher in set specimens compared with fresh specimens (P<0.05).

After 72 hours:

The percentage of viability of cells treated by different concentrations of Pulpine Mineral in both fresh and set forms had no significant difference with each other but was significantly lower than that of the control group by approximately 60% (P<0.05).

### Results of the MTT assay for Pulpine NE cytotoxicity

After 24 hours:

The viability percentage of cells treated with different concentrations of fresh and set Pulpine NE significantly decreased by approximately 80% after 24 hours compared with the control group (P<0.05). Cell viability and proliferation were more favorable only in cells exposed to ¼ diluted extract of set Pulpine NE, compared with other groups.

After 72 hours:

The viability percentage of cells treated with different concentrations of fresh and set Pulpine NE had no significant difference with each other but was significantly lower than the control group by approximately 50-70% (P<0.05). The percentage of cell viability was higher in ¼ diluted extract of set Pulpine NE than in other groups (P<0.05).

### Comparison of the cytotoxicity of MTA, Pulpine Mineral, and Pulpine NE after 24 hours

Fresh form:

As indicated in Figure-1A, the reduction in cell viability percentage was significantly greater following exposure to pure and diluted extracts of Pulpine Mineral and Pulpine NE compared with MTA (P<0.05). The difference in cell viability was not significant following exposure of cells to pure and ½ diluted extracts of Pulpine Mineral and Pulpine NE (P>0.05). However, in ¼ diluted extracts, the percentage of cell viability was significantly higher in Pulpine Mineral compared to Pulpine NE (P<0.05).

Set form:

As shown in Figure-1B, the percentage of cell viability was significantly higher following treatment with pure extract of MTA compared with Pulpine Mineral and Pulpine (P<0.05); also, this rate in Pulpine Mineral was significantly higher than that in Pulpine NE (P<0.05). The difference in cell viability was not significant following exposure to ½ and ¼ diluted extracts of MTA and Pulpine Mineral (P>0.05); however, the cell viability percentage in both MTA and Pulpine Mineral groups was significantly higher than that in Pulpine NE (P<0.05).

### Comparison of the cytotoxicity of MTA, Pulpine Mineral, and Pulpine NE after 72 hours

**Figure-2:**
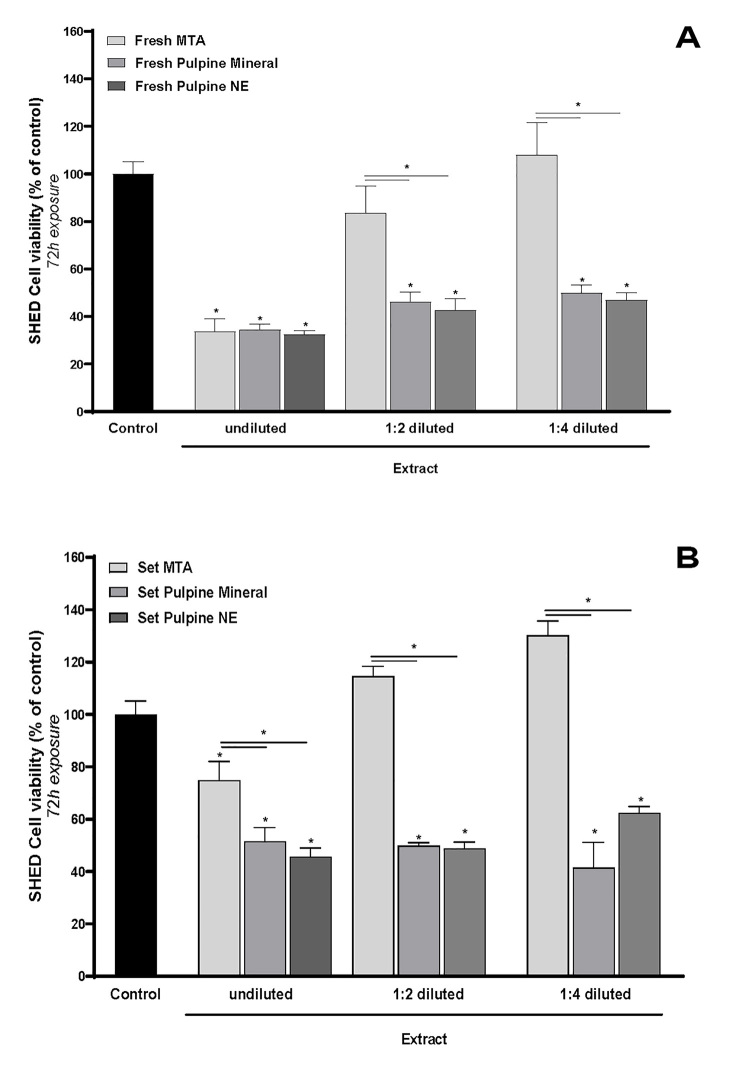
Figure 2. Comparison of the cytotoxicity of MTA, Pulpine Mineral, and Pulpine NE extracts after 72 hours in fresh (A) and set (B) forms. Starts on top of the columns indicate presence of a significant difference with the control group, and starts on the lines indicate presence of a significant difference between the two groups (P< 0.05)

Fresh form:

As indicated in Figure-2A, no significant difference in cell viability was found among the pure extracts of the three groups (P>0.05). The ½ and ¼ diluted extracts of Pulpine Mineral and Pulpine NE had no significant difference in cell viability (P>0.05); however, they both showed significantly lower cell viability than MTA (P<0.05).

Set form:

As shown in -ure-2B, pure and diluted extracts of Pulpine Mineral and Pulpine NE had no significant difference in cell viability (P>0.05); however, they both showed significantly lower cell viability in all three concentrations compared with MTA (P<0.05).

### Migration of SHEDs following exposure to ¼ diluted extracts of set MTA, Pulpine Mineral, and Pulpine NE for 0, 24, and 48 hours

**Figure-3:**
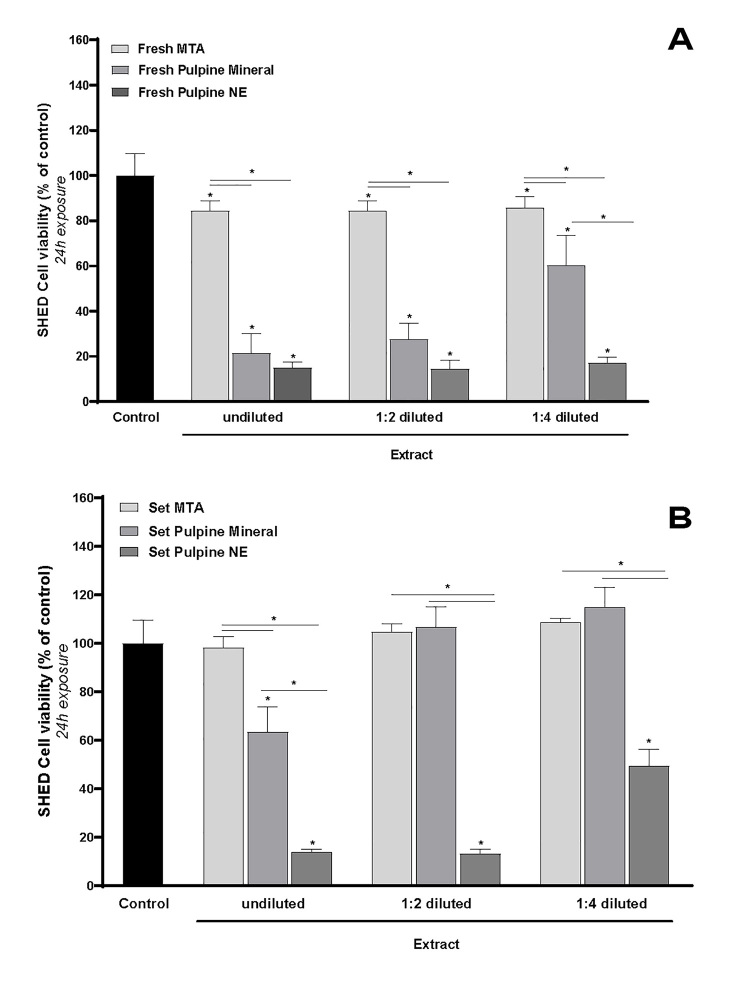
Figure 3. Micrographs of the migration of SHEDs following exposure to ¼ diluted extracts of set MTA, Pulpine Mineral, and Pulpine NE for 0, 24, and 48 hours at x40 magnification compared to the control group

As illustrated in Figure-3, cell migration in the first 24 hours was not significantly different between the MTA and control groups. Cell migration in the Pulpine Mineral group was lower than that in the MTA and control groups; however, all cells were dead and there was no proliferation or migration in the Pulpine NE group.

At 48 hours after exposure, the scratch was completely closed with 100% cell migration in the control and MTA groups, and cell density was even higher in the MTA group than in the control group. Cell migration was noted in the Pulpine Mineral group, and the scratch was slightly filled compared to 24 hours; however, healing was not complete. All cells were dead and there was no proliferation or migration in the Pulpine NE group.

### Qualitative SEM assessment of the morphology and adhesion of SHEDs to the surface of MTA, Pulpine Mineral, and Pulpine NE specimens

**Figure-4:**
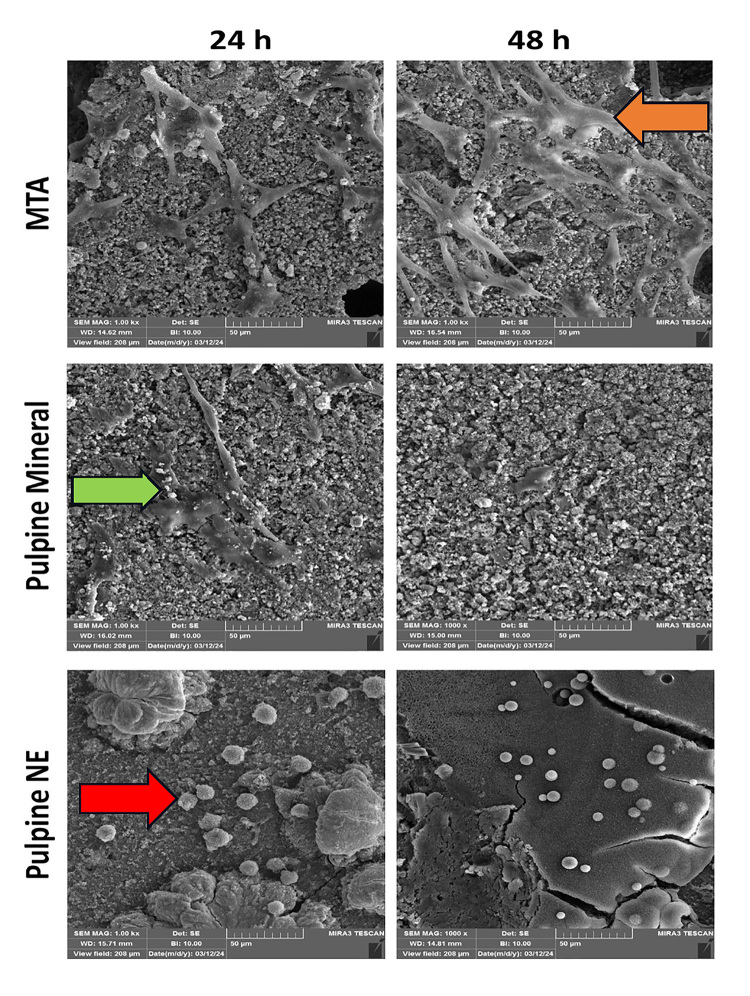
Figure 4. SEM micrographs of SHEDs following exposure to MTA, Pulpine Mineral, and Pulpine NE in the scratch test., Attached cells with pseudopods are seen in the MTA group (orange arrow). Round and apoptotic cells are noted on Pulpine NE specimens (red arrow). Small number of cells with pseudopods are seen on Pulpine Mineral specimens (green arrow).

As shown in Figure-4, cells showed optimal adhesion in the MTA group at 24 and 48 hours. All cells were dead in the Pulpine NE group with no adhesion. Some cells were seen adhering to the surface of Pulpine NE specimens after 24 hours; however, they decreased in number after 48 hours.

In the MTA groups, attached cells with pseudopods were seen (orange arrow in Figure-4). However, round and apoptotic cells were noted on Pulpine NE specimens (red arrow in Figure-4). Small number of cells with pseudopods were seen on Pulpine Mineral specimens that decreased in number after 48 hours (green arrow on Figure-4).

## Discussion

This study appears to be the first to assess the effects of Pulpine Mineral and Pulpine NE on the viability, proliferation, adhesion and migration of stem cells from SHEDs, in comparison with MTA.

With respect to cytotoxicity of the materials, the obtained results showed that Pulpine NE in both fresh and set forms significantly decreased cell viability at both 24 and 72 hours compared with MTA; however, set Pulpine Mineral in ½ and ¼ concentrations had no significant difference with MTA at 24 hours. Cells showed higher viability when exposed to set form of MTA compared with fresh form, which is because of the fact that fresh material is leachable. In line with the present results, Ghoddusi et al. [19] showed higher time-dependent cytotoxicity of fresh materials, compared with set form. Torshabi et al. [20] pointed to higher cytotoxicity of MTA and calcium-enriched mixture cement in fresh form, which increased with time; while the cytotoxicity of set forms decreased with time. In contrast to the present results, Camilleri et al [21]. reported higher biocompatibility and lower cytotoxicity of freshly mixed MTA compared with its set form. However, Saidon et al. [22] demonstrated that freshly mixed MTA caused denaturation of adjacent cells and proteins, and was surrounded by an area of lysed cells, with normal cells behind this area. After complete setting, the pH changed, decreasing cell damage. An animal study on dogs found no significant difference in cementum formation between freshly mixed and set MTA although the latter had lower cytotoxicity; thus, it appears that small differences between fresh and set forms reported under in vitro and in vivo conditions do not have clinical significance [23].

In the present study, cell viability increased with time following exposure to diluted extracts, compared to pure form, irrespective of set or fresh form. Similarly in the clinical setting, byproducts of the setting reaction are diluted in tissue fluids and are eliminated by the blood flow, which is known as the washout effect [24][25]. However, this biological response is not present in vitro. In line with the present results, Rodrigues et al. [26] reported that the viability of human dental pulp stem cells increased by a reduction in concentration of the MTA extract. Reduction of cytotoxicity by dilution of extracts has been reported in several other studies as well [27][28][29][30]. Gradual release of hydroxyl ions (and increased alkalinity) may decrease cell viability in vitro; however, they are neutralized by tissue fluids in the clinical setting. Fung et al. [31] reported a significant increase in proliferation of SHEDs following a reduction in propolis concentration. The molecular mechanism behind the cytotoxicity of propolis has not been well elucidated. Flavonoids are among the main constituents of propolis, which is an active pharmaceutical agent and a strong anti-oxidant. Also, propolis is extracted by alcohol-based and non-alcohol-based extraction methods. Future studies are required to extract propolis using a non-alcoholic solvent since previous studies on alcoholic extract of propolis showed its cytotoxicity [12][32][33].

To the best of the authors’ knowledge, no previous in vitro study is available on Pulpine Mineral and Pulpine NE, and only some animal studies are available in this respect. A previous animal study assessed the histopathological and immunohistochemical responses of dental pulp of immature teeth in dogs to Pulpine Mineral and Pulpine NE compared with MTA. MTA yielded the best results; cell viability was lower in Pulpine Mineral than MTA but with no statistical significance. However, the results of Pulpine NE were significantly lower than Pulpine Mineral and MTA [16]. Superior results of Pulpine Mineral compared to Pulpine NE may be due to the fact that Pulpine Mineral contains hydroxyapatite crystals in addition to propolis, and may contribute to a higher success rate because fibroblasts release alkaline phosphatase following exposure to hydroxyapatite. Alkaline phosphatase is an osteoinductive material for differentiation of progenitor cells [34]. In contrast, Pulpine NE was not successful in dentinal bridge formation. Presence of zinc compounds in its composition may be responsible for the unfavorable response since they can be both genotoxic and cytotoxic [35]. Zinc added to Pulpine NE may affect matrix metalloproteinases and suppress or decelerate pulp recovery as such [36]. Bastawy et al, [37] also compared Biodentine and Pulpine NE as pulp-capping agents in dogs’ teeth and showed the superiority of Biodentine in dentinal bridge formation, tissue organization, and anti-inflammatory effects. They attributed this difference to their different chemical composition, mechanical properties, and sealability. Pulpine NE is a calcium-based material with a low concentration of calcium hydroxide [37]. Due to the solubility of calcium hydroxide and continuous release of calcium and hydroxyl ions, this material has a constantly stimulating effect while Biodentine is a calcium silicate-based cement and has a high ion release in the initial setting, which is later diminished, providing a suitable environment for pulp recovery [38][39].

Scratch test results in the present study revealed complete closure and healing of the scratch in the control and MTA groups with 100% cell migration after 48 hours; cell density was even higher in the MTA group than in the control group. Partial closer and cell migration were noted at 24 hours in the Pulpine Mineral group; however, all cells were dead in the Pulpine NE group. In the clinical setting, dental pulp stem cells proliferate, migrate to the site of injury, and differentiate into odontoblasts in deeply carious cavities with a residual dentin thickness of 0.01 to 0.25 mm or in case of pulpal exposure [40]. Cell migration is imperative to preserve tissue homeostasis and regeneration [41]. Thus, dental materials should enhance cell migration. Collado-González et al. [42] reported optimal cell migration 48 hours after exposure to Biodentine and MTA extracts especially in 1:4 concentration while IRM and TheraCal LC did not induce the migration of SHEDs. Consistent with the present results, several studies reported optimal cell migration in response to treatment with MTA Angelus [43][44][45].

SEM assessment of cell adhesion in the present study revealed optimal adhesion in the MTA group at both 24 and 48 hours while all cells were dead in the Pulpine NE group. A few adhered cells with pseudopods were noted in the Pulpine Mineral group after 24 hours, which decreased in number after 48 hours. Cell adhesion is a reliable criterion for evaluation of the biological effects of bioactive materials since adhesion is required prior to cell proliferation and differentiation, and secretion of mineralized extracellular matrix. A previous study reported that presence of spindle-shaped cells with extensive pseudopods is a good indicator of cell viability adjacent to the respective material [46]. Collado-González et al. [42] reported high adhesion of cells with pseudopods in the form of an extensive layer to MTA Angelus and Biodentine, which was in line with the present observations.

The obtained results in the Pulpine NE group may be due to the presence of zinc and calcium hydroxide in its powder, and the resultant chronic irritation. Also, zinc has cytotoxic effects and can activate matrix metalloproteinases [35][36]. Additionally, superior results in the Pulpine Mineral group may be due to the presence of hydroxyapatite in its composition, which is not present in Pulpine NE [34]. Inferior results of Pulpine Mineral and Pulpine NE compared to MTA can be due to the fact that MTA is a calcium silicate-based cement, which provides optimal conditions for pulp recovery while Pulpine Mineral and Pulpine NE are based on calcium hydroxide [37][39]. Also, propolis is present in the composition of their liquid component, which contains flavonoids and has acidic effects. It appears that concentration of Pulpine Mineral and Pulpine NE is an important parameter in determining the vital pulp tissue response [12][31][32][33].

Caffeiic acid phenethyl ester (CAPE), one of the active ingredients in propolis, has the ability to either promote or inhibit stem cell proliferation based on its concentration.By boosting the expression of TGF-beta, a growth regulating factor that can promote the proliferation of stem cells, CAPE can increase cellular proliferation in areas where propolis dosage is lower. However, a larger concentration of CAPE causes cell death, suggesting that a higher concentration of propolis may disrupt cell homeostasis and cause the P53 protein to be expressed, which in turn triggers apoptosis and accelerates cell death [47].

Before evaluating the effectiveness of novel dental materials in animal models or clinical trials, laboratory investigations are frequently employed in research to characterize and create a biological profile of the materials. These concentrations might not be the same as those employed in biological or clinical settings, even though they offer thorough details on the substance being studied and adhere to laboratory standards.As a result, the findings of these investigations need to be interpreted more carefully. The byproducts of pulp capping material reactions are probably diluted in intracellular fluids and removed through the blood vessels in clinical settings within the human body. The washout effect that takes place in in vivo settings is demonstrated by the removal of dental materials that come into touch with living tissue. Laboratory studies, however, do not exhibit this physiologic response. The concentration of the constituent compounds of the two recently introduced materials is a crucial point that is not covered in the brochures because of their commercial character. To evaluate the pulp’s reaction to Pulpine Mineral and Pulpine NE’s ingredients, more research with distinct designs is needed. The in vitro approach of this study restricts how broadly the results may be applied.

This study had an in vitro design, which limits the generalizability of the findings. Future in vitro studies are required to find other properties of these materials such as cell differentiation. Also, animal studies and clinical trials are required to compare their clinical success.

## Conclusion

Pulpine Mineral

showed superior biological effects compared to Pulpine NE; however, both Pulpine groups exhibited inferior results compared to MTA Angelus.

## Conflict of Interest

The author declares that they have no competing interests.
